# Efficacy of Digital Speech Therapy for Poststroke Dysarthria: Randomized Noninferiority Trial

**DOI:** 10.2196/81938

**Published:** 2026-05-18

**Authors:** Yuyoung Kim, Minjung Kim, Saebyeol Kim, Jinwoo Kim, Joon-Ho Shin, Yoonkyung Chang, Ji Young Na, JungWan Kim, Tae-Jin Song

**Affiliations:** 1 HAII Corp., Seoul, Republic of Korea; 2 HCI Lab, Yonsei University, Seoul, Republic of Korea; 3 Department of Neurorehabilitation, National Rehabilitation Center, Seoul, Republic of Korea; 4 Department of Neurology, Ewha Womans University College of Medicine, Mokdong Hospital, Seoul, Republic of Korea; 5 Department of Communication Disorders, Korea Nazarene University, Cheonan, Republic of Korea; 6 Department of Speech and Language Pathology, Daegu University, Gyeongsan, Republic of Korea; 7 Department of Neurology, Ewha Womans University Seoul Hospital, Ewha Womans University College of Medicine, Seoul Hospital, 260 Gonghang-daero, Gangseo-gu, Seoul, 07804, Republic of Korea, 82 1522-7000; 8 Graduate Program in System Health Science and Engineering, Ewha Womans University, Seoul, Republic of Korea

**Keywords:** stroke, dysarthria, speech therapy, mobile health app, mHealth, randomized clinical trial

## Abstract

**Background:**

Poststroke dysarthria, a common speech impairment, affects up to half of all stroke survivors, often reducing their ability to communicate, and adversely affecting their quality of life. Although conventional speech therapy for poststroke dysarthria is effective, access is often limited by time and geographical constraints. Here, digital speech therapy may serve as a remotely deliverable alternative for selected patients. However, few trials have assessed its efficacy, safety, and usability.

**Objective:**

This study aimed to evaluate whether a smartphone-based speech therapy app is noninferior to conventional workbook-based therapy in improving speech intelligibility among individuals with poststroke dysarthria.

**Methods:**

This single-blind, randomized controlled, noninferiority trial was performed at 3 hospitals in South Korea. Adults (≥19 y) with poststroke dysarthria who were cognitively intact, without aphasia, and able to use a smartphone were eligible. Participants were enrolled between July 20, 2023, and April 15, 2024. Participants were randomly assigned (1:1), stratified by stroke phase, using a block randomization method, to receive either a smartphone-based digital therapy app or a conventional workbook-based therapy for 4 weeks. The primary outcome was speech intelligibility (0‐100 perceptual rating) after the intervention. Primary analysis was intention-to-treat using analysis of covariance. A noninferiority margin of 19 points was pre-defined.

**Results:**

A total of 73 participants were enrolled (median age 62.00 years). Among them, 38 were assigned to the digital speech therapy group and 35 to the control group. Intelligibility scores improved from 80.48 (SD 18.92) to 92.08 (SD 12.38) in the intervention group, and from 80.94 (SD 16.74) to 88.11 (SD 18.06) in the control group. The adjusted between-group difference was 4.49 (95% CI 0.61-8.37), and the lower bound of the 95% CI was above the prespecified noninferiority margin (–19), which supported noninferiority. No significant between-group differences were observed in the secondary outcomes related to speech function or psychological status. The system usability score was 89.6, and adherence in the digital speech therapy group was 64.6% based on app logs, with no treatment-related adverse events.

**Conclusions:**

Digital speech therapy was noninferior to conventional workbook-based therapy in improving speech intelligibility and was feasible across acute to early subacute and chronic stroke phases in cognitively intact stroke survivors with predominantly mild-to-moderate dysarthria. However, feasibility and efficacy in older stroke survivors with cognitive deficits or co-occurring aphasia, or in those unable to use smartphones, remain to be established.

## Introduction

Stroke is a leading cause of death and long-term disability worldwide [1]. Approximately 22%‐60% of stroke survivors present with motor speech disorders, including poststroke dysarthria. Additionally, approximately 35% of patients with poststroke dysarthria continue to experience dysarthria after 6 months [2-5]. These findings indicate that poststroke dysarthria is a common and persistent complication of stroke, typically characterized by minimal long-term recovery. Poststroke dysarthria can involve impairments in articulation, phonation, prosody, resonance, and respiration [3]. Moreover, poststroke dysarthria may compromise speech intelligibility and potentially have a detrimental impact on communication, emotional well-being, and the overall quality of life [6,7].

Behavioral speech therapy is the standard treatment for poststroke dysarthria. It involves structured exercises designed to strengthen respiratory and articulatory muscles, improve motor speech control, and enhance intelligibility through targeted modulation of pitch, loudness, and articulation [3,8]. Despite its proven benefits, conventional face-to-face speech therapy remains difficult to access because of practical limitations. Its repetitive and monotonous nature contributes to poor long-term adherence [9]. In addition, access to trained speech-language pathologists (SLPs) is often limited, and patients with poststroke dysarthria experience challenges visiting hospitals or speech therapy clinics multiple times per week [10,11]. Therefore, these patients often face an unmet need for access to adequate speech therapy.

Digital speech therapy has recently emerged as a promising alternative to address the limitations of conventional treatments. Mobile health apps and computer-based programs enable the delivery of personalized speech therapy outside of hospitals. These digital speech therapies allow patients to engage in high-frequency and intensive practice with reduced need for real-time clinician involvement [12,13]. Previous studies suggest that telerehabilitation and digital platforms can reduce the burden and cost of face-to-face speech therapy [14,15]. They also help maintain continuity of care after hospital discharge and encourage patients to practice independently [14-16]. These advantages make digital speech therapy particularly beneficial for stroke survivors who have difficulty accessing conventional speech therapy owing to geographic, financial, or pandemic-related reasons.

Previous small-sample studies have suggested potential benefits of digital speech therapy for individuals with poststroke dysarthria in the chronic phase [17-19]. However, limited sample sizes and a lack of rigorous evaluation imply that the efficacy and safety of these interventions remain unclear. Consequently, we performed a multicenter randomized controlled trial to determine whether smartphone-based digital therapy is noninferior to conventional workbook-based therapy in improving speech intelligibility in patients with poststroke dysarthria. We also evaluated secondary outcomes, including speech function, patient-reported psychological well-being, usability, and adherence, to assess the broader therapeutic impact and overall feasibility.

## Methods

### Study Design

This multicenter, prospective, evaluator-blinded, randomized controlled trial used a noninferiority design to compare a smartphone-based digital speech therapy app with conventional workbook-based therapy in patients with poststroke dysarthria. The study was conducted at 3 stroke centers in South Korea, including Ewha Womans University Seoul Hospital, Ewha Womans University Mokdong Hospital, and the National Rehabilitation Center in Seoul.

The trial was registered at ClinicalTrials.gov (NCT05865106). The study protocol outlined the intervention development, outcome measure selection, and a previously published statistical analysis plan [20].

### Participants

Participants were recruited between July 20, 2023 and April 15, 2024, across the 3 participating sites in South Korea. Stroke specialists screened patients in stroke units and outpatient clinics at each of the 3 hospitals. The inclusion criteria were as follows: (1) adults aged 19 years or older; (2) diagnosis of dysarthria caused by stroke, confirmed by a stroke specialist; (3) dysarthria severity corresponding to a score of 1 or higher on the dysarthria item of the National Institutes of Health Stroke Scale [21], reflecting either mild-to-moderate dysarthria, where speech is slurred but mostly intelligible, or severe dysarthria, where speech is mostly unintelligible or absent, and aphasia had to be absent; (4) a neurologically stable condition with no evidence of acute deterioration; (5) first-ever stroke with no prior history of stroke; (6) mini-mental state examination score of 26 or higher, which was used to confirm the absence of cognitive impairment in the participants [22]; (7) sufficient visual acuity to read therapy materials [23]; (8) adequate hearing to follow spoken instructions [24]; and (9) sufficient motor skills and basic communication ability to use a smartphone. The participants had to be able to press buttons and understand simple operations on the device.

The exclusion criteria were as follows: (1) presence of aphasia or other language disorders; (2) other neurological conditions that could affect speech, such as Parkinson disease, Parkinsonism, or motor neuron disease; (3) major psychiatric illness, which included schizophrenia, untreated major depression, or a history of substance abuse that might interfere with participation; (4) unwillingness or inability to use a smartphone or the speech therapy app; (5) inability to read or understand task instructions; and (6) inability to speak or understand Korean, as the intervention materials were written and spoken in Korean. Biological sex was recorded as male or female based on medical records at enrollment.

All participants provided written informed consent before enrollment in the study. They were informed that they could withdraw from the study at any time without affecting their standard medical care. The research team could also withdraw participants under certain conditions. These included completion of less than 40% of the assigned therapy sessions, emergence of urgent medical problems, adverse events related to the intervention, noncompliance with study procedures, or clinical deterioration that made continued participation unsafe.

### Randomization and Masking

Participants were randomly assigned in a 1:1 ratio to either the digital speech therapy or control group. Randomization was stratified according to the stroke phase to ensure that each group included a balanced number of participants in the acute to early subacute and chronic phases. The acute to early subacute phase was defined as less than 6 months after stroke onset. The chronic phase was defined as 6 months or more after stroke onset [25].

Randomization sequences for each stratum were generated using a computer-based algorithm in R software (R Foundation for Statistical Computing). Block sizes of 2 and 4 were used to ensure allocation concealment and unpredictability [26]. An independent researcher who was not involved in the trial prepared the randomization schedule and managed the group assignment process. The allocation was concealed until all baseline assessments were completed. Each participant was assigned a sequential study identification number at enrollment, after which group assignment was performed based on the next entry in the corresponding stratified randomization list. The participants and treating clinicians were informed of the group assignment only after the baseline evaluation was completed.

Owing to the nature of the interventions, blinding of the participants and treating clinicians was not feasible. Therefore, all speech recordings were centrally collected after trial completion and anonymized. The filenames and metadata contained no group, site, or time point identifiers and were presented in a computer-generated random order to independent, trained SLP assessors. These assessors had no role in the treatment, had no contact with the participants, and were blinded to treatment assignment, study site, and assessment timepoint. This approach ensured objective evaluation of the primary outcomes despite the open-label design of the trial [27].

### Procedures

Following randomization, the therapy phase began within one week. Participants in both groups engaged in their assigned therapy program for 4 weeks. Sessions were performed 5 days per week, with each session lasting approximately 60 minutes. A trained research coordinator provided a standardized orientation session to explain how to use the assigned therapy tools, either the mobile app or printed workbook. All the participants were instructed to complete the therapy independently. Caregiver assistance was permitted, if necessary, in both the digital speech therapy and workbook-based control groups.

During the intervention period, the study staff contacted the participants 3 times per week via phone or SMS text messaging. These check-ins were intended to monitor progress, encourage continued participation, and resolve any difficulties related to the exercises, such as technical problems or misunderstandings. In addition to these contacts, no in-person therapy or direct clinical input was provided. Participants completed all exercises independently at home without the direct involvement of a clinician.

Participants completed outcome assessments at baseline and after completing the 4-week intervention. All assessments were performed in person during scheduled hospital visits using a standardized smartphone-based app.

### Intervention Group: Smartphone-Based Speech Therapy App

Participants assigned to the intervention group received therapy through a smartphone-based speech therapy app developed by our team in collaboration with clinical experts. The app was specifically designed for individuals with poststroke dysarthria and incorporated the established principles of behavioral speech therapy [3,28].

At the start of the intervention, an app was installed on the smartphone of each participant (Android or iOS). A research coordinator performed an in-person orientation session to guide the participants in using the app, and a printed instruction manual was also provided. The participants were instructed to complete 60 minutes of therapy daily, 5 days per week, for 4 weeks. The participants were permitted to divide the session into smaller time blocks depending on their preference and fatigue level.

Digital speech therapy was individualized based on the baseline assessments. Recorded speech samples of the participants were uploaded to a secure server after enrollment. A licensed SLP reviewed these samples and performed an auditory-perceptual evaluation targeting 5 key subsystems typically affected in dysarthria, including respiration, phonation, resonance, articulation, and prosody [3]. Based on this assessment, the SLP created a personalized therapy plan. This plan prescribed the initial starting level and weekly goals. The app implemented the SLP’s prescription by delivering the specified exercise sets. The app did not automatically adapt difficulty in real time. Adjustments were made by the SLP during weekly remote check-ins.

The app included 15 structured exercises organized into the following four therapeutic categories: (1) warm-up tasks using video-guided instructions to establish correct posture and breathing patterns; (2) phonation and prosody tasks, such as sustained vowel production and modulation of pitch and loudness, with real-time visual feedback to enhance vocal control; (3) oro-motor and vocal strength exercises that included tongue, lip, and jaw movements and vocal fold training via video-guided routines to strengthen articulatory and phonatory coordination; and (4) articulation exercises involving repetition of words and sentences, as well as aloud reading tasks that emphasized clarity, speech rate, and prosodic control. Specifically, the app visualized pitch variation and loudness in real time, and at the end of each task, it provided a summary indicating how closely the performance matched the preset target range.

The app automatically recorded usage data, including task completion, number of attempts, and exercise duration. Adherence was defined as the percentage of completed sessions out of the 20 prescribed sessions (5 sessions per week for 4 weeks). Individual adherence scores were derived by dividing the number of sessions completed by the 20 prescribed sessions and expressing the result as a percentage (0%‐100%). Participants could view their progress on a dashboard within the app. The research team monitored the data remotely using a secure web-based portal. When adherence dropped below expectations, study staff, independent of treatment and outcome assessment, proactively contacted participants to provide encouragement or technical assistance. This system supported consistent speech therapy delivery without in-person clinician supervision while maintaining engagement throughout the intervention. A detailed description of digital speech therapy for poststroke dysarthria is provided in the Multimedia Appendix 1.

### Control Group: Conventional Speech Therapy Workbook

Participants assigned to the control group received a conventional paper-based speech-therapy workbook. The workbook was based on standard clinical practices and evidence-based protocols for poststroke dysarthria and included structured exercises used in conventional therapy.

The workbook was organized to reflect the general content and progression of the app to ensure structural comparability with digital speech therapy, which included similar types of exercises and daily dosages. It featured activities targeting key speech subsystems, such as syllable and sentence repetition, sustained vowel phonation, volume and pitch control, and expressive reading tasks involving prosodic variation. The workbook followed a standardized stepwise sequence from easier to more challenging exercises. Participants proceeded in order and were instructed to complete the same daily and weekly dose as in the intervention group. The overall level of difficulty and therapeutic intensity matched those of the digital therapy program. The workbook provided static written instructions, and participants were instructed to self-monitor their performance without real-time feedback; no therapeutic performance feedback was delivered during the home practice sessions.

Before beginning the program, a licensed SLP provided standardized instructions on using the workbook. Participants were instructed to complete 60 minutes of therapy per day, 5 days per week, for 4 weeks. A printed log sheet was provided to track session completion and to note any challenges encountered.

To promote adherence and ensure treatment fidelity, a study coordinator contacted participants 3 times per week over phone or SMS text messaging. During these check-ins, the coordinator encouraged practice and reviewed self-reported task completion. Adherence was monitored using participants’ self-reported log entries and verbal confirmation during follow-up contacts. Consistent with the intervention group, adherence was calculated as the percentage of self-reported sessions completed relative to the 20 prescribed sessions.

The control group participants were offered access to digital speech therapy for poststroke dysarthria after completing the study to ensure ethical equity. Those who wanted to participate were granted access to the same 4-week digital speech therapy program. A detailed description of the workbook-based speech therapy protocol is provided in the Multimedia Appendix 1.

### Outcomes

To ensure comparability, all efficacy outcome instruments, recording workflows, and rater procedures were identical for both groups. Secondary and patient-reported efficacy measures were also administered using the same protocols in both groups.

The primary outcome was speech intelligibility, defined as the extent to which a listener could accurately understand the utterances of the speaker [29]. Participants read the Gaeul Passage, a standardized Korean text comprising 369 syllables with a balanced phonemic distribution [30]. Speech samples were recorded using the study app and securely stored on a remote server. Three independent, licensed SLPs with ≥3 years of experience in adult poststroke dysarthria assessment served as a fixed evaluation panel. Each rater evaluated every participant recording once and provided an independent perceptual speech intelligibility rating on a 0‐100 scale. The participant’s final speech intelligibility score was the mean of the 3 rater scores [31]. The interrater reliability was assessed using the intraclass correlation coefficient (ICC) based on a 2-way random-effects model with absolute agreement to ensure the appropriateness of using an average score. The average-measures ICC was 0.85 (95% CI 0.74-0.91).

Secondary outcomes included measures of speech function and psychological well-being. Speech function was assessed using the following measures: (1) maximum phonation time (MPT), defined as the duration for which participants could sustain the vowel /a/ [32]; (2) oral diadochokinesis, measured by the number of correctly repeated syllables (/pa/, /ta/, /ka/, /pataka/) within a 5-second interval and normalized to syllables per second [33]; and (3) percentage of consonants correct (PCC), evaluated using the urimal test of articulation and phonology-2, in which participants read 30 Korean words and their productions were transcribed and compared with the target forms [34,35]. To ensure objectivity and eliminate rater bias, MPT and diadochokinesis were automatically calculated by the digital platform’s software for both groups. PCC was evaluated by a licensed SLP blinded to both treatment allocation and assessment time points.

Psychological well-being was assessed using the Patient Health Questionnaire-9 (PHQ-9), a 9-item self-report scale for depressive symptoms [36], with lower scores indicating reduced symptom severity. The quality of life of dysarthric speakers (QoL-Dys) comprises 4 domains (speech characteristics, situational difficulty, compensatory strategies, and perceived reactions of others); lower scores indicate improved quality of life with less functional impact from dysarthria. The total QoL-Dys score was used for analysis [37,38].

Usability was evaluated using the System Usability Scale (SUS), a 10-item self-report questionnaire that assesses perceived ease of use and user satisfaction [39]. This measure was administered only to participants in the intervention group at the end of the 4-week trial. Safety was monitored through weekly check-ins with all participants, and any adverse events were recorded by the study team.

### Statistical Analysis

The sample size estimation determined that 76 participants (38 per group) would provide >80% power to assess the noninferiority of the primary outcome. This calculation assumed an SD of 24.9, a noninferiority margin of 19 points on the 100-point speech intelligibility scale, and a one-sided α of .025. A noninferiority margin of 19 points on the 0‐100 speech intelligibility scale was defined. This margin was based on clinical judgment regarding what would constitute an acceptable difference in functional communication in routine home practice. This rationale reflects the established clinical perspective that speech intelligibility scores should be interpreted within the context of functional communication rather than as isolated numerical values [40]. A 19-point noninferiority margin, anchored in a functional communication framework, was applied to the task-based primary outcome derived from standardized passage reading (Gaeul Passage). Recent estimates of the minimal clinically important difference for intelligibility (approximately 7‐15 points) were considered as contextual benchmarks [41]. Given the limited availability of stroke-specific guidance for defining an intelligibility-based margin, the prespecification was anchored to a functional-communication framework and informed by methodological recommendations that noninferiority margins should reflect clinical acceptability and the objective of the trial [42]. Practical considerations regarding remote, self-guided delivery motivated the use of a noninferiority design; however, the noninferiority margin was determined based on clinical acceptability.

All analyses were performed using IBM SPSS Statistics version 29.0 (IBM Corp). Interim analyses were not performed. The Shapiro-Wilk test was used to assess the normality of continuous variables. As most variables were nonnormally distributed, baseline comparisons used the Mann-Whitney *U*-test for continuous variables and the *χ*² test or Fisher exact test for categorical variables. Descriptive statistics are presented as medians with IQR or counts with percentages.

Outcomes were analyzed using analysis of covariance (ANCOVA), with baseline scores and age entered as covariates. Given the baseline age imbalance, we performed exploratory analyses to verify the directionality and influence of the age adjustment. Details are provided in Table S3 in Multimedia Appendix 2. The model assumptions, including residual normality, were verified and satisfied. Adjusted mean differences (intervention minus control), 95% CIs, *P* values, and partial eta-squared values were reported. Noninferiority was concluded when the 95% CI lay entirely above the margin of –19 points.

The primary analysis was conducted on the intention-to-treat (ITT) population, which included all randomized participants. To handle missing postintervention data, the last observation carried forward method was applied (baseline carried forward). In addition, a complete-case sensitivity analysis excluding participants with missing postintervention outcomes was performed. Additionally, a per-protocol (PP) analysis was conducted on a set comprising participants who adhered to the protocol (≥70% adherence). Because adherence assessment differed between groups (objective app logs vs self-reported checklists), PP classification, particularly in the control arm, should be interpreted cautiously.

To assess robustness, further sensitivity analyses were conducted to evaluate the treatment effect under different assumptions. These included: (1) a subgroup analysis by stroke phase (acute to early subacute vs chronic) with an interaction test; (2) an ANCOVA that additionally adjusted for stroke phase as a covariate; (3) a ranked ANCOVA (Quade test) to account for nonnormality in baseline scores; and (4) a logit-transformed ANCOVA to model intelligibility on its original bounded scale.

This study did not include a data monitoring committee, as it was a short-term behavioral intervention trial with minimal risk.

### Ethical Considerations

The protocol was approved by the Institutional Review Boards of all participating centers (approval numbers: EUMC 2023-02-002 for Ewha Womans University Seoul Hospital and Ewha Womans University Mokdong Hospital; NRC-2023-01-007 for the National Rehabilitation Center), and the study was conducted in accordance with the Declaration of Helsinki [43]. All participants provided written informed consent before enrollment. Participants received KRW 25,000 (approximately US $19.20) per visit. The study included 2 visits, for a total of KRW 50,000 (the conversion was based on an exchange rate of US $1=KRW 1300 at the time of the study). Study data were deidentified before analysis, and no identifiable participant data were included. Audio files were anonymized and stored on secure servers, with access restricted to authorized researchers.

## Results

### User Statistics

Of 90 patients screened for eligibility, 17 were excluded for the following reasons: 5 did not meet the inclusion criteria (including n=3 with uncorrected visual impairment and n=2 unable to use smartphone technology), 9 declined to participate, and 3 were excluded due to relocation or inability to attend follow-up visits.

**Figure 1.**
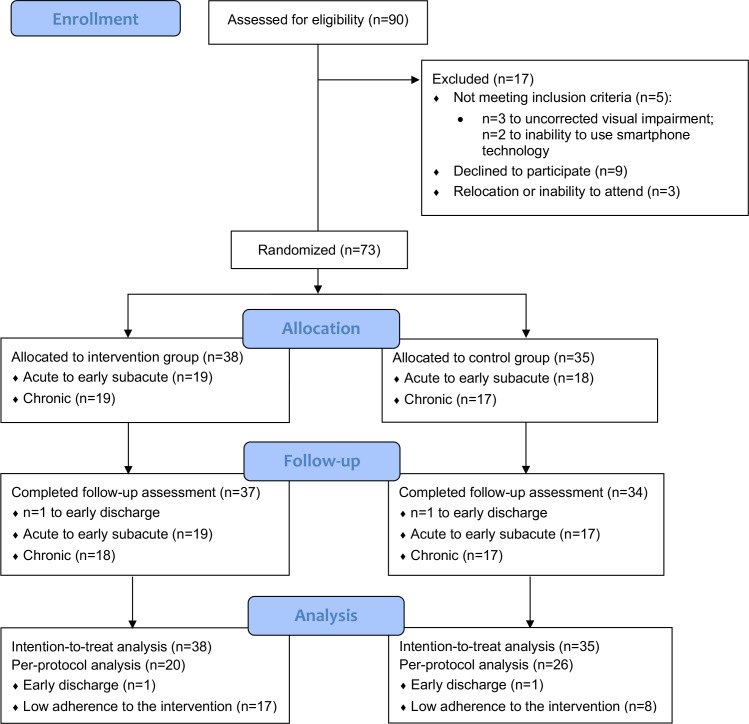
CONSORT (Consolidated Standards of Reporting Trials) diagram.

A total of 73 participants were enrolled and randomized, with 38 assigned to the smartphone-based digital therapy group and 35 assigned to the conventional workbook-based therapy group. Two participants (one from each group) discontinued the intervention. Consequently, the primary ITT analysis included all 73 randomized participants, and the PP population comprised 46 participants. These participants were included in the final analysis. Figure 1 shows the trial profile.

The baseline characteristics are summarized in Table 1. Baseline speech intelligibility was high (median 89.00%), and most participants were classified as having mild-to-moderate dysarthria (66/73, 90%). The baseline characteristics were generally comparable between the groups, although statistically significant between-group differences were observed for age (*P*=.03) and baseline diadochokinesis /pataka/ rate (*P*=.02).

**Table 1.** Baseline demographic and clinical characteristics of the participants.

Participant characteristics	Total (n=73)	Intervention (n=38)	Control (n=35)	*P* value
Sex, n (%)				.83
Male	53 (73)	28 (74)	25 (71)	
Female	20 (27)	10 (26)	10 (29)	
Age (years), median (IQR)	62.00 (50.50‐70.50)	59.50 (48.75‐67.25)	64.00 (55.50‐75.50)	.03
Phase of stroke, n (%)				.90
Acute to early subacute	37 (51)	19 (50)	18 (51)	
Chronic	36 (49)	19 (50)	17 (49)	
Duration after stroke (days), median (IQR)	66.00 (11.50‐796.50)	128.50 (12.00‐606.75)	66.00 (11.50‐956.50)	.67
Acute to early subacute	12.00 (7.50‐22.50)	12.00 (7.00‐19.00)	11.50 (8.00‐39.75)	.57
Chronic	796.50 (368.75‐2139.00)	636.00 (343.00‐1875.00)	975.00 (725.50‐2388.50)	.32
mRS^a^, median (IQR)	1.00 (1.00‐2.00)	2.00 (1.00‐2.00)	1.00 (1.00‐2.00)	.31
Total NIHSS^b^, median (IQR)	3.00 (2.00‐5.00)	4.00 (3.00‐6.00)	3.00 (2.00‐5.00)	.15
NIHSS^b^_dysarthria, median (IQR)	1.00 (1.00‐2.00)	1.00 (1.00‐2.00)	1.00 (1.00‐2.00)	.09
Dysarthria severity, n (%)				.11
Mild to moderate	66 (90)	32 (84)	34 (97)	
Severe	7 (10)	6 (16)	1 (3)	
Type of stroke, n (%)				.53
Cerebral infarction	65 (89)	33 (87)	32 (91)	
Cerebral hemorrhage	8 (11)	5 (13)	3 (9)	
Speech intelligibility, median (IQR)	89.00 (77.67‐90.67)	89.33 (77.67‐92.17)	89.00 (76.67‐90.00)	.26
PHQ-9^c^, median (IQR)	5.00 (3.00‐9.00)	5.00 (2.00‐9.00)	5.00 (3.00‐9.00)	.52
QoL-Dys^d^, median (IQR)	63.00 (47.50‐81.00)	63.00 (50.50‐74.50)	65.00 (46.00‐89.00)	.55
MPT^e^ /a/, median (IQR)	6.60 (3.47‐10.70)	6.61 (3.73‐9.21)	6.59 (2.88‐10.81)	.69
PCC^f^ (%), median (IQR)	98.00 (94.00‐100.00)	99.00 (94.00‐100.00)	98.00 (94.00‐99.00)	.07
Diadochokinesis^g^ /pa/, median (IQR)	4.22 (3.25‐5.10)	4.65 (3.49‐5.26)	3.83 (3.05‐4.82)	.08
Diadochokinesis^g^ /ta/, median (IQR)	4.52 (3.31‐5.31)	4.71 (3.33‐5.47)	4.21 (3.23‐4.86)	.19
Diadochokinesis^g^ /ka/, median (IQR)	3.93 (2.74‐4.80)	3.95 (3.18‐4.73)	3.81 (2.66‐4.83)	.59
Diadochokinesis^g^ /pataka/, median (IQR)	1.30 (0.97‐1.64)	1.43 (1.07‐1.83)	1.18 (0.93‐1.51)	.02

a mRS: modified Rankin scale.

b NIHSS: National Institutes of Health Stroke Scale.

c PHQ-9: Patient Health Questionnaire-9.

d QoL-Dys: Quality of Life in the Dysarthric Speaker.

e MPT: maximum phonation time.

f PCC: percentage of consonants correct.

g Diadochokinesis rate reported as syllables per second over a 5-second interval.

### Evaluation Outcomes

**Figure 2.**
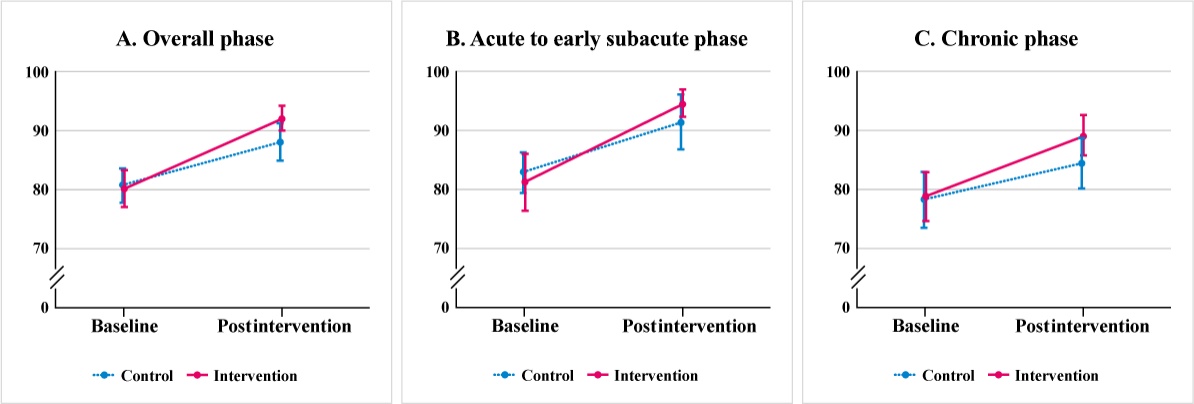
Speech intelligibility outcomes. (A) Speech intelligibility scores at baseline and postintervention for the intervention and control groups across all participants (score range: 0‐100); (B) Speech intelligibility scores for participants in the acute to early subacute stroke phase; (C). Speech intelligibility scores for participants in the chronic stroke phase; error bars represent standard errors.

The primary outcome results are summarized in Table 2 and Figure 2A. Speech intelligibility improved in both groups after the intervention. In the intervention group, the mean score increased from 80.48 (SD 18.92) to 92.08 (SD 12.38), whereas in the control group, it increased from 80.94 (SD 16.74) to 88.11 (SD 18.06). In the ANCOVA model adjusting for baseline intelligibility and age, the adjusted mean difference in postintervention intelligibility was 4.49 (95% CI 0.61-8.37). Because the lower bound of the 95% CI exceeded the prespecified noninferiority margin of –19, noninferiority was concluded. A complete-case sensitivity analysis excluding the 2 participants with missing postintervention outcomes (n=71) yielded a similar adjusted mean difference of 4.41 (95% CI 0.62-8.20), consistent with the primary analysis. The mean within-group improvement in the control group was 7.17 points, corresponding to the lower threshold of the minimal clinically important difference range [41].

**Table 2.** Adjusted speech intelligibility outcomes, per protocol analysis and subgroup analyses based on analysis of covariance (ANCOVA).

Group	Baseline, mean (SD)	Postintervention, mean (SD)	Estimated difference, MD^a^ (95% CI)	*F*^b^ (df)	*P* value	*η²* ^c^
Overall			4.49 (0.61 to 8.37)	5.32 (1, 67)	.02	0.07
Intervention	80.48 (18.92)	92.08 (12.38)				
Control	80.94 (16.74)	88.11 (18.06)				
Acute to early subacute			3.79 (–2.14 to 9.72)	1.69 (1, 32)	.20	0.05
Intervention	81.35 (20.89)	94.71 (10.05)				
Control	83.37 (13.84)	91.45 (18.43)				
Chronic			4.74 (–0.24 to 9.73)	3.76 (1, 31)	.06	0.11
Intervention	78.61 (17.27)	89.45 (14.51)				
Control	78.37 (19.45)	84.57 (17.51)				
PP analysis^d^			3.57 (–1.79 to 8.92)	1.80 (1, 42)	.19	0.04
Intervention	78.03 (21.01)	91.10 (13.91)				
Control	77.99 (18.58)	87.01 (20.83)				

a MD: mean difference.

b *F*: F statistic

c *η*²: partial eta square (effect size estimate)

d PP: per-protocol (≥70% adherence)

**Figure 3.**
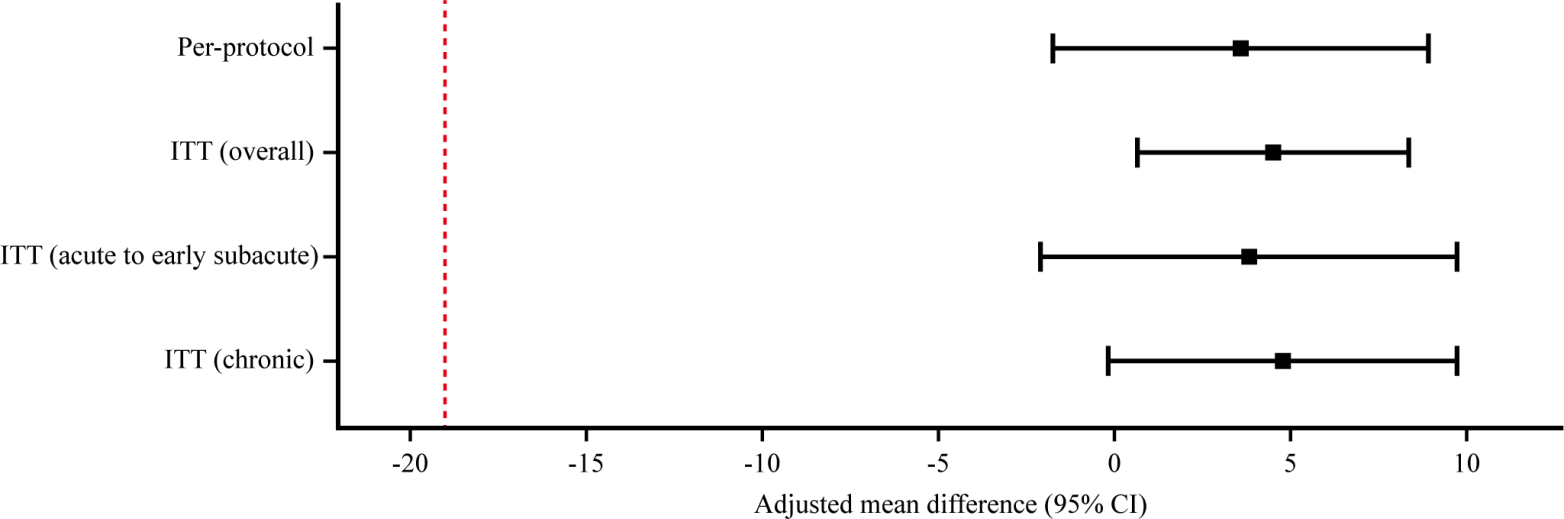
Noninferiority analysis of the primary outcome adjusted mean differences (intervention minus control) with 95% CIs are shown for the intention-to-treat (ITT), per-protocol (PP), and ITT subgroup analyses by stroke phase. The dashed vertical line indicates the prespecified noninferiority margin (–19), and the solid vertical line indicates no difference. ITT: intention-to-treat.

In the per-protocol set (n=46), defined as participants with ≥70% adherence, the adjusted mean difference in postintervention intelligibility was 3.57 (95% CI –1.79 to 8.92). Adherence was assessed using app usage logs in the intervention arm and self-reported checklists in the control arm. The lower bound of the confidence interval also remained above the non-inferiority margin, consistent with the primary noninferiority finding. Figure 3 summarizes these noninferiority results.

The subgroup and sensitivity analyses supported the robustness of the primary noninferiority conclusion. Subgroup analyses stratified by stroke phase yielded similar adjusted mean differences in both strataFigure 2B and 2C. Among participants in the acute to early subacute phase, the adjusted mean difference in intelligibility scores was 3.79 (95% CI –2.14 to 9.72). In the chronic group, the difference was 4.74 (95% CI –0.24 to 9.73). In both strata, the lower bounds of the 95% CIs remained above the prespecified noninferiority margin (–19). The treatment-by-phase interaction test showed no evidence of differential effects by stroke phase. A sensitivity analysis including stroke phase as a covariate yielded a similar adjusted difference of 4.51 (95% CI 0.71-8.31), which supported the consistency of the primary noninferiority finding. We also performed a ranked ANCOVA (Quade’s test) to address nonnormality in baseline distributions, which yielded inferences consistent with the primary analysis. Finally, a logit-transformed ANCOVA was conducted to account for the bounded 0%‐100% outcome; the adjusted group effect was of similar magnitude and direction (estimated mean difference 0.52 on the logit scale; 95% CI –0.01 to 1.05), with inference consistent with the primary analysis. Full numerical results, which include exploratory analyses examining age adjustment (Table S3) and assay sensitivity and robustness checks (Table S4), are provided in Multimedia Appendix 2.

Secondary outcomes showed no statistically significant differences between the groups in speech production, psychological status, or QoL-Dys measures. A detailed summary of the secondary outcomes is presented in Table 3. For speech function, the adjusted between-group difference in MPT was 0.82 (95% CI –1.26 to 2.91; *P*=.43), whereas in PCC it was 0.14 (95% CI –1.82 to 2.10; *P*=.88). No significant group differences were observed in the diadochokinesis syllable repetition rates for /pa/, /ta/, /ka/, or /pataka/. Patient-reported psychological outcomes also exhibited no significant differences between the groups. The adjusted difference in PHQ-9 scores was –1.02 (95% CI –3.16 to 1.13; *P*=.35). Similarly, total scores on the QoL-Dys scale did not differ significantly between groups (–3.04; 95% CI –14.72 to 8.65; *P*=.61). No significant differences were observed in any of the 4 QoL-Dys subdomains.

**Table 3.** Secondary outcome measures.

Group	Baseline, mean (SD)	Postintervention, mean (SD)	Estimated difference, MD^a^ (95% CI)	*F*^b^ (df)	*P* value	*η²* ^c^
PHQ-9^d^			−1.02 (−3.16 to 1.13)	0.89 (1, 67)	.35	0.01
Intervention	5.89 (4.49)	5.29 (3.77)				
Control	6.77 (5.11)	6.66 (5.64)				
QoL-Dys^e^ total			−3.04 (−14.72 to 8.65)	0.27 (1, 67)	.61	0.00
Intervention	61.92 (21.23)	57.97 (23.08)				
Control	63.97 (30.25)	63.43 (30.89)				
Speech characteristics			−0.33 (−3.44 to 2.77)	0.05 (1, 67)	.83	0.00
Intervention	17.61 (6.08)	16.42 (6.73)				
Control	19.20 (8.54)	17.51 (7.76)				
Situational difficulty			−2.02 (−5.47 to 1.43)	1.37 (1, 67)	.25	0.02
Intervention	16.29 (7.85)	14.24 (7.16)				
Control	17.69 (10.46)	17.34 (9.03)				
Compensatory strategies			0.31 (−3.18 to 3.81)	0.03 (1, 67)	.86	0.00
Intervention	17.34 (6.14)	16.42 (5.58)				
Control	15.63 (8.19)	15.91 (8.73)				
Perceived reactions of others			−1.54 (−4.02 to 0.94)	1.53 (1, 67)	.22	0.02
Intervention	10.68 (5.80)	10.89 (7.54)				
Control	11.46 (8.37)	12.66 (7.79)				
PCC^f^ (%)			0.14 (−1.82 to 2.10)	0.01 (1, 67)	.88	0.00
Intervention	96.18 (7.64)	96.61 (6.95)				
Control	95.43 (5.40)	95.97 (6.11)				
MPT^g^			0.82 (−1.26 to 2.91)	0.62 (1, 65)	.43	0.01
Intervention	7.95 (5.71)	9.12 (5.33)				
Control	7.06 (4.31)	7.52 (5.30)				
Diadochokinesis /pa/^h^			0.11 (−0.37 to 0.59)	0.19 (1, 65)	.66	0.00
Intervention	4.28 (1.44)	4.62 (1.08)				
Control	3.82 (1.33)	4.20 (1.33)				
Diadochokinesis **/**ta/^h^			0.39 (−0.03 to 0.80)	3.40 (1, 65)	.07	0.05
Intervention	4.39 (1.32)	4.81 (1.28)				
Control	4.02 (1.31)	4.22 (1.09)				
Diadochokinesis /ka/^h^			0.20 (−0.20 to 0.60)	1.01 (1, 65)	.32	0.01
Intervention	3.83 (1.11)	4.07 (1.24)				
Control	3.70 (1.30)	3.76 (1.24)				
Diadochokinesis /pataka/^h^			0.03 (−0.15 to 0.21)	0.12 (1, 65)	.74	0.00
Intervention	1.44 (0.45)	1.61 (0.52)				
Control	1.17 (0.41)	1.35 (0.47)				

a MD: mean difference.

b *F*: F statistic

c *η*²: partial eta square (effect size estimate).

d PHQ-9: Patient Health Questionnaire-9.

e QoL-Dys: Quality of Life in Dysarthria.

f PCC: percentage of consonants correct.

g MPT: maximum phonation time.

h Diadochokinesis: rate reported as syllables per second over a 5-second interval.

The usability of the digital application was only assessed in the intervention group. The mean usability score was 89.57 (SD 10.70). In the intervention group, participants completed a mean of 64.6% of the prescribed sessions according to objective application logs. The control group reported a mean adherence of 74.3% via self-reported checklists. In both arms, these rates represent the group average of individual completion percentages relative to the 20 prescribed sessions. Because adherence was assessed using different methods across groups (objective logs vs self-report), these adherence rates are reported descriptively within each arm and should not be directly compared.

### Safety and Adverse Events

No serious adverse events related to either the digital therapy or the conventional workbook were reported in either group. Regarding nonserious events, a few participants in the intervention group experienced minor technical issues, such as login difficulties or brief application errors. These issues were promptly resolved by the research team and did not interfere with continued intervention use. No other intervention-related adverse events, such as vocal strain, fatigue, or headache, were reported by participants in either group.

## Discussion

### Principal Findings

This randomized controlled trial evaluated whether a smartphone-based speech therapy app could achieve therapeutic outcomes comparable to those of conventional workbook-based therapy in patients with poststroke dysarthria. Our findings suggest that remotely delivered, self-guided speech therapy may be a feasible alternative to the workbook-based home program used in this trial, with remote monitoring and minimal clinician contact. The primary ITT analysis met the prespecified noninferiority criterion, and this finding was supported by the PP analysis and sensitivity analyses. The robustness of the primary results was further supported by a nonparametric analysis accounting for nonnormal baseline distributions, which yielded consistent directional findings. Although the point estimate was in the direction of benefit for the intervention, the trial was designed and powered for noninferiority; therefore, we interpret the findings within the prespecified noninferiority framework and consider any suggestion of superiority as exploratory. Interpretation of noninferiority also depends on assay sensitivity, which assumes that the active control would be expected to demonstrate efficacy in the trial setting. As highlighted in the International Council for Harmonisation of Technical Requirements for Pharmaceuticals for Human Use (ICH) E10 guideline, if the comparator effect is attenuated, noninferiority findings may be more difficult to interpret. In the present sample, the mean improvement in the workbook-based control arm was 7.17 points, which aligns with the lower bound of the cited minimal clinically important difference range [41]; this should be considered when interpreting the strength of the noninferiority inference [44].

The effectiveness of speech therapy for poststroke dysarthria may vary depending on the stroke phase. In the early phase, patients are believed to benefit more from restorative approaches that leverage the heightened neuroplasticity shortly after injury [45,46]. By contrast, those in the chronic phase typically rely on compensatory strategies to optimize their residual abilities [47]. However, most previous clinical trials have focused on a single phase of stroke, most commonly the chronic phase. For instance, clinician-delivered programs such as Lee Silverman Voice Treatment and the “Be Clear” protocol have demonstrated improvements in articulatory precision, loudness, and speech clarity but were limited to participants in the chronic phase [18,19]. In contrast, our previous pilot study focused exclusively on patients in the acute to early subacute phases and revealed improvements in speech intelligibility with the use of a digital speech therapy application [48].

Given that most previous studies have targeted a single stroke phase, the applicability of digital speech therapy across different stroke phases has not yet been well established. By including individuals in the acute to early subacute and chronic phases, this trial suggests the potential to broaden the evidence base for its use across the various stroke phases.

Stratified analyses revealed no statistically significant interaction between the treatment group and stroke phase, indicating no evidence of differential treatment effects by phase. This is of particular interest given the widely cited hypothesis that the early poststroke phase represents a sensitive period of heightened neuroplasticity and responsiveness to therapy [49,50]. Contrary to this expectation, our data revealed that participants in the chronic phase exhibited numerically greater gains in speech intelligibility with digital speech therapy than the control group, whereas the acute to early subacute phase exhibited a smaller difference. Although this trend did not reach statistical significance, it may reflect differences in engagement or responsiveness, which warrant further exploration.

Several factors could explain this pattern. Patients in the chronic phase may have a greater awareness of their communication limitations and stronger motivation to improve them. Long-term adaptation to dysarthria can foster clearer personal goals and greater readiness to engage in structured therapy [51]. Moreover, the chronic phase may offer a more neurologically stable condition that enables consistent training and facilitates the acquisition of compensatory strategies. In contrast, individuals in the early phases may still manage co-occurring impairments and undergo spontaneous recovery, making it more difficult to concentrate on speech-specific interventions. Taken together, these findings suggest that although the stroke phase is an important consideration, patient-level factors, such as motivation, engagement, and adherence, may also contribute to therapeutic outcomes, particularly in the chronic phase. This interpretation aligns with previous studies indicating that, even in the chronic phase, individuals can achieve functional gains when provided with structured and accessible interventions [18,29]. These observations suggest that future studies should consider both stroke phase and individual characteristics when evaluating response to therapy. Effective interventions should consider both the stroke phase and individual characteristics, such as motivation, baseline severity, and access to support resources [52]. Tailored strategies may help optimize recovery regardless of whether the therapy is delivered during the early or chronic phase. This interpretation was further reinforced by a covariate-adjusted analysis, which confirmed the consistency of the treatment effects regardless of the stroke phase. Additionally, since the acute to early subacute subgroup was enrolled very early after stroke onset (median 12 d), spontaneous neurological recovery likely contributed to a portion of the improvements observed in both the intervention and control groups. At this early stage, rapid natural recovery may have attenuated between-group differences and masked treatment-specific effects, making it difficult to disentangle the intervention’s effect from natural recovery. Therefore, gains in this stratum should be interpreted cautiously and not attributed solely to the digital or workbook-based therapy.

The secondary outcomes exhibited no statistically significant differences between the intervention and control groups. Measures related to phonation (MPT), articulation (PCC and diadochokinesis), and patient-reported psychological outcomes (PHQ-9 and QoL-Dys) exhibited similar postintervention scores in both groups without meaningful between-group differences. The intervention group exhibited modestly higher mean values in some domains; for instance, a 1.05-second longer MPT. However, these differences did not reach statistical significance. These findings are in line with those of previous studies reporting that digital and conventional speech therapies may offer similar benefits in improving speech-motor control and psychosocial functioning when delivered with adequate structure and intensity [51].

Participants in the digital speech therapy group rated the usability of the app highly (mean SUS score of 89.57). On average, they completed 64.6% of the prescribed sessions based on system usage logs. The observed 64.6% adherence rate in the intervention group is consistent with findings from other self-managed rehabilitation programs. For context, completion rates in stroke rehabilitation studies range from approximately 61% to 100%, and broader exercise adherence reviews commonly report mid-range rates of 60%‐80% [53,54]. In the control group, adherence was monitored using self-report checklists and follow-up contacts. Because adherence in the control arm was based on self-report, these records should be interpreted descriptively and should not be directly compared with objectively logged adherence in the digital arm. While reasons for nonadherence were not systematically collected, informally reported barriers included participant fatigue, environmental constraints (eg, the need for a quiet space for vocal practice), and challenges with digital literacy, particularly among older adults. The high usability ratings and observed engagement in the digital arm were in line with those of previous studies on digital health in neurology and rehabilitation [14]. Features such as real-time visual feedback, simple navigation, and self-paced home practice were well-received. These findings echo earlier reports indicating that stroke survivors appreciate the autonomy and flexibility offered by well-designed mobile therapy tools [14,15,55]. Collectively, these results underscore the feasibility of digital therapy within the selected cohort enrolled in this trial, which included older participants who were neurologically stable, cognitively intact, and able to use a smartphone. Thus, this modality may offer a promising option for selected patients with limited access to in-person treatment. Nevertheless, the applicability of these findings is limited to the selected cohort enrolled in this trial.

### Limitations

Despite these promising findings, this study had several limitations. Although the sample size was sufficient to assess noninferiority of the primary outcome, it may have been underpowered to detect smaller differences in secondary outcomes or to fully explore subgroup effects. In addition, the trial randomized 73 participants, which was slightly below the planned sample size of 76, and this minor shortfall should be considered when interpreting the statistical power of the study.

The generalizability of our findings is limited by the sample characteristics. We enrolled cognitively intact participants without aphasia or other language disorders, required basic smartphone proficiency, and restricted the trial to native Korean speakers. While these criteria ensured feasibility and task compliance, they may limit external validity for individuals with cognitive or linguistic impairments, older adults with low digital literacy, or non-Korean speakers. Crucially, the feasibility and efficacy of this digital intervention remain unproven in stroke survivors with cognitive deficits, co-occurring aphasia, or significant multimorbidity, as the eligibility criteria excluded these populations or required a level of digital proficiency that may not be present in the broader stroke population. Future studies should include more diverse populations and culturally/linguistically adapted versions of the intervention to enhance applicability across settings.

The study assessed treatment outcomes only immediately after the 4-week intervention, and no follow-up evaluation was conducted to examine sustained efficacy. Consequently, the long-term maintenance of treatment gains remains uncertain. It is unclear whether the observed improvements would persist without continued use of the program or periodic reinforcement. Future research should include extended follow-up periods to evaluate the durability of effects and to determine optimal dosing schedules or maintenance strategies for long-term benefit.

Because this was a multicenter trial, we standardized the comparator condition across sites by compiling commonly used conventional practice components into this workbook-based home program to ensure protocol consistency. However, this format may not have fully captured clinically relevant elements of routine face-to-face speech therapy, particularly SLP-delivered feedback and individualized real-time cueing. Future trials could consider combining standardized in-person therapy with structured workbook-based home practice in the control condition to strengthen the comparator further while preserving protocol consistency across centers. Although both groups received comparable study contact, the standardized workbook progression without performance-based adjustment may have resulted in less individualized dosing than the intervention arm.

Adherence in the control group was assessed using self-report checklists, which may be subject to reporting variability. Accordingly, we did not perform a formal between-group comparison of adherence or explore adherence as a moderator of treatment effects. This difference in adherence monitoring introduced measurement uncertainty and limited the ability to verify the comparability of therapeutic effort between groups. Notably, using self-reported data to define the PP population in the control arm may have led to an overestimation of engagement relative to the digital arm. Consequently, readers should exercise caution when interpreting adherence-based analyses, and the noninferiority findings should be considered in the context of this potential reporting bias in the conventional therapy group. Future studies should implement more structured or objective adherence tracking methods for nondigital arms, such as time-stamped photo diaries or scripted phone check-ins with specific queries about session time.

There was also a difference in personalization between groups. The intervention group followed an SLP-prescribed plan, whereas the control group used a standardized stepwise workbook program with a fixed progression and no performance-based advancement rules. Although both groups received comparable monitoring and guidance, this difference in personalization may have favored the intervention group. Future studies should include a workbook arm with SLP-tailored prescriptions to balance personalization across modalities. In addition, usability was assessed only in the intervention group to evaluate the technical feasibility of the app, a core trial objective. Since the SUS is designed specifically for digital interfaces, it was not applied to the paper-based control. This difference prevented a direct comparison of satisfaction between modalities, which limited conclusions on relative clinical acceptability. Future research should use generalized measures to enable more robust comparisons across different treatment formats.

Our study had limitations regarding measurement sensitivity. The baseline intelligibility score was high (median 89.00%). While supplementary analysis suggested that pronounced ceiling compression was uncommon (eg, one participant scored above 95%; Figure S5 in Multimedia Appendix 2), the high baseline level may have constrained measurable improvement. This may have reduced the sensitivity of the primary outcome measure to detect change and made between-group differences harder to detect. In addition, because the primary outcome was derived from a standardized passage reading, the ratings may partly reflect articulatory precision and familiarity with the passage rather than functional intelligibility in everyday communication. Consequently, a limitation exists in that our noninferiority margin, although anchored in a functional communication framework, was applied to a more structural, task-based measure. Therefore, the 19-point margin may carry different clinical weight in this standardized context compared with spontaneous or conversational speech, and readers should be cautious in extrapolating these findings directly to real-world communicative success. This limitation should be considered when interpreting the clinical implications of the noninferiority margin and the primary outcome [56,57]. Using the same passage at baseline and postintervention may also introduce practice effects despite the 4-week interval. Familiarity with standardized assessment materials among trained raters may have contributed to higher scores. Future studies should strengthen measurement sensitivity by incorporating spontaneous speech or conversation-based evaluations, using multiple assessment materials, and including multiple raters. Accordingly, the present findings most directly apply to cognitively intact stroke survivors with mild-to-moderate dysarthria, and additional studies are needed to establish efficacy in patients with more severe impairment.

Although the baseline characteristics were generally well-balanced between the groups, a statistically significant difference in age was observed. Older age is often associated with slower motor recovery and reduced neuroplasticity, which may introduce confounding factors into the interpretation of treatment effects. Notably, the intervention group was younger and thus potentially more responsive to therapy. This imbalance should be considered when interpreting the comparative effectiveness of the interventions.

### Conclusions

This randomized controlled trial evaluated whether a smartphone-based speech therapy app was noninferior to conventional workbook-based therapy in improving speech intelligibility in cognitively intact adults with poststroke dysarthria. Digital speech therapy achieved a level of improvement comparable to that of the control group, which met the predefined noninferiority margin. These findings support the clinical potential of remotely delivered self-guided speech therapy for stroke survivors with mild-to-moderate dysarthria, while the modest mean improvement observed in the workbook-based control arm should be considered when interpreting the strength of the noninferiority inference. Further evaluation is needed for patients with more severe impairment. Future studies should assess the long-term sustainability of treatment effects, explore integration into routine clinical workflows, and evaluate the effectiveness across various languages and health care systems.

## Supplementary material

10.2196/81938 Multimedia Appendix 1 Details of smartphone-based and workbook-based therapy.

10.2196/81938 Multimedia Appendix 2 Supplementary results including detailed outcome measures and additional analyses.

10.2196/81938 Checklist 1 CONSORT-EHEALTH (v 1.6.1) checklist.

## Abbreviations

ANCOVA: analysis of covariance
CONSORT: Consolidated Standards of Reporting Trials
ICC: intraclass correlation coefficient
ITT: intention-to-treat
MPT: maximum phonation time
PCC: percentage of consonants correct
PHQ-9: Patient Health Questionnaire-9
PP: per-protocol
QoL-Dys: Quality of Life in the Dysarthric Speaker
SLP: speech-language pathologist
SUS: System Usability Scale
