# Supplementary material for: Efficacy of Digital Speech Therapy for Poststroke Dysarthria: Randomized Noninferiority Trial
Source: J Med Internet Res. 2026 May 18;28:e81938. doi: 10.2196/81938 (PMC13182877; doi:10.2196/81938)
Supplement: Multimedia Appendix 1 [file jmir-v28-e81938-s001.docx]

## Multimedia Appendix

## Details of Smartphone-Based and Workbook-Based Therapy

The smartphone-based speech therapy application used in this trial was developed to provide individualized, intensive treatment for patients with post-stroke dysarthria. The intervention consisted of three sequential modules: (1) speech assessment, (2) therapist-guided treatment planning, and (3) self-administered home-based exercises (Figure S1).


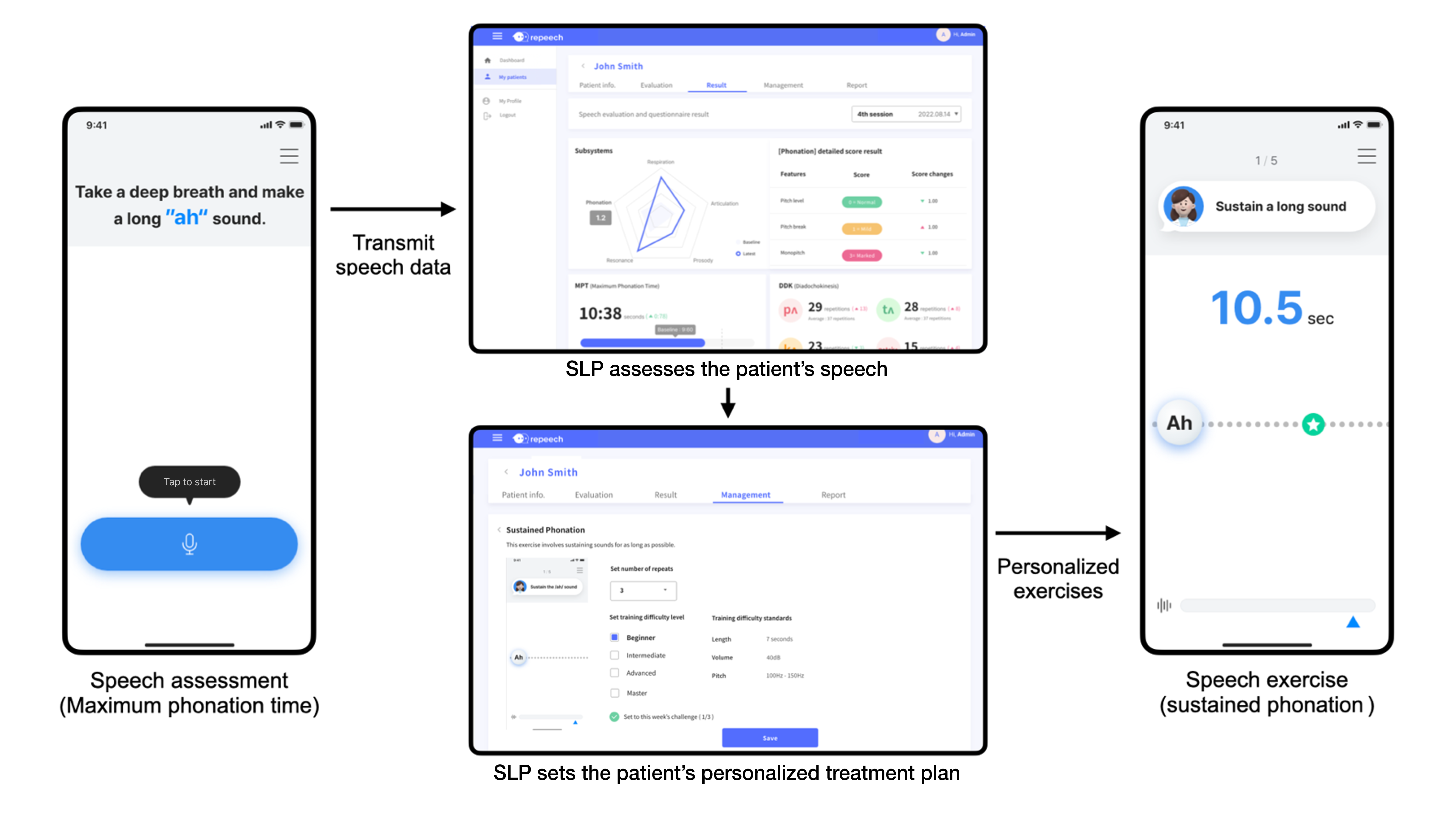


Figure S1. Structure of the digital speech therapy system. Patients complete assessment and training tasks via the application. Clinicians review recordings on a secure web dashboard and generate personalized training plans.

### 1. Speech Assessment Tasks

Before initiating treatment, participants completed four standardized speech tasks using the mobile application. These tasks were designed to assess key speech subsystems commonly used in dysarthria. Recordings were uploaded automatically and reviewed by licensed speech-language pathologists (SLPs) via a secure web dashboard.

**Maximum Phonation Time (MPT):** Assessed phonatory control and respiratory support. Participants sustained the vowel “ah” as long as possible. Duration was calculated automatically.

**Diadochokinetic (DDK) Syllable Repetition:** Measured articulatory speed and coordination by counting repetitions of /pa/, /ta/, /ka/, and /pataka/ within a 5-second interval.

**Word Reading Task:** Evaluated segmental articulation by prompting participants to read aloud a list of 30 phonetically balanced Korean words (UTAP-2).

**Passage Reading (Gaeul Passage):** Measured overall intelligibility, prosody, and connected speech. Participants read a standardized 369-syllable Korean passage aloud.

Each task was guided by both auditory and written instructions and performed in a quiet environment to ensure recording quality.

### 2. Therapist-Guided Treatment Planning

SLPs reviewed the uploaded assessment data through a dedicated web dashboard. Based on perceptual analysis of the recordings, SLPs evaluated speech error patterns and coordination domains. They then prescribed personalized training regimens, customizing task types, sequencing, difficulty levels, and repetition counts for each patient. These settings were remotely delivered to the patient’s application.

### 3. Home-based Exercises

Participants in the intervention group completed 60-minute self-guided training sessions, five days per week, for four weeks. Each session followed a fixed progression of tasks to reinforce motor learning and included the following components:

Training Tasks: The exercises in the application were designed to target key speech domains, which include phonation, articulation, prosody, and respiratory support. These tasks were guided by auditory and visual instructions and structured to gradually increase in difficulty based on weekly performance data reviewed by the therapist. Specifically, the intervention consisted of the following 15 types of exercises:

- Posture correction
- Oral motor exercises
- Sustained phonation
- Gliding up
- Gliding down
- Pitch control
- Volume up
- Effortful closure exercises
- Yawn–sigh exercises
- Contrast drills
- Contrastive stress
- Breathing exercises
- Tapping
- Slow reading
- Loud reading

**Real-Time Visual Feedback:** During each task, an interactive interface provided continuous visual feedback on vocal loudness, pitch, and speed. Users were instructed to adjust their voice in real time to match target parameters. After completing each task, users were shown a summary screen displaying their performance results. Accuracy was classified into three levels based on the match between their output and target values. The system also allowed users to replay their recorded voice to self-monitor performance and track progress over time.

**Customization and Adaptation:** Initial difficulty levels were based on baseline assessment results. Parameters such as decibel thresholds and pitch targets were adjusted weekly by the SLP during remote check-ins.

**Monitoring and Logging:** The application automatically records task completion and usage adherence. Participants conducted all exercises independently without real-time interaction with a clinician.

## Description of the Workbook-Based Speech Therapy

Participants in the control group received a printed workbook titled *Speech Therapy Workbook for Dysarthria*, which was developed by licensed SLPs based on established in-person treatment protocols. The workbook was designed to enable self-administered, home-based speech training and mirrored the structure and therapeutic goals of the digital application.

The program consisted of daily training over four weeks, with participants encouraged to complete approximately 60 minutes of exercises per day. Clear written instructions and visual illustrations were provided for each task. A training checklist at the end of the workbook allowed participants to track adherence on a daily basis. Tasks could be performed independently or with a caregiver’s assistance, and the use of a mirror was recommended for visual feedback when appropriate.

| 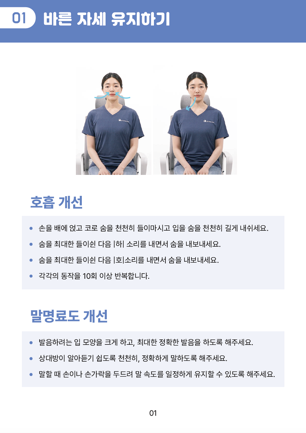 | 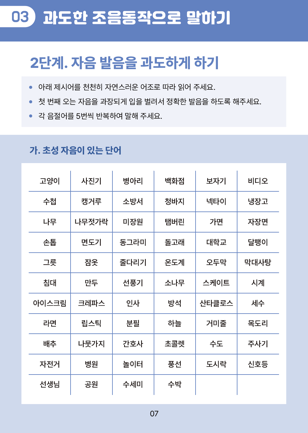 | 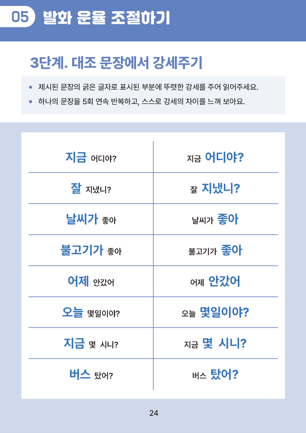 | 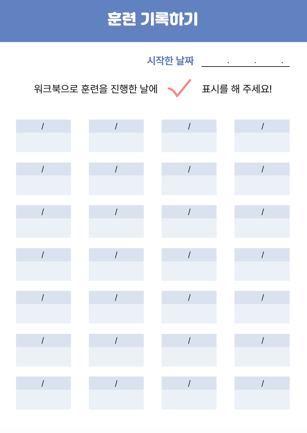 |
| --- | --- | --- | --- |

Figure S2. Examples of workbook content and the adherence checklist. Tasks shown include posture training for breath control, over-articulation of consonants, contrastive stress within sentences, and the self-check grid for daily tracking. Each page includes simple instructions and visual guidance to support user understanding and consistency.

Workbook exercises targeted core speech subsystems, which include phonation, articulation, resonance, prosody, and respiratory coordination. The training tasks included:

- Postural alignment and breathing support
- Facial and oral motor warm-up
- Vowel prolongation with controlled airflow
- Syllable and word articulation drills (e.g., initial consonant emphasis)
- Word pair contrast exercises based on place/manner of articulation
- Sentence repetition and connected speech practice
- Intonation and emphasis adjustment using sentence types
- Structured reading aloud with pacing and pausing cues
- Rhythm and stress variation for semantic clarity
- Spontaneous speech generation with thematic prompts
