# Supplementary material for: Efficacy of Digital Speech Therapy for Poststroke Dysarthria: Randomized Noninferiority Trial
Source: J Med Internet Res. 2026 May 18;28:e81938. doi: 10.2196/81938 (PMC13182877; doi:10.2196/81938)
Supplement: Multimedia Appendix 2 [file jmir-v28-e81938-s002.docx]

## Multimedia Appendix 2.

## Supplementary results

To verify the robustness of the main findings, a series of complementary analyses was conducted. These analyses were designed to ensure that the primary conclusions regarding speech intelligibility were not artifacts of specific statistical models, recovery stages, or demographic imbalances. The results across all supplementary tests were directionally consistent with the primary intention-to-treat analysis, which supports the stability of the treatment effect.

### 1. Robustness of Treatment Effects (Sensitivity Analyses)

The primary analysis was supplemented by several sensitivity analyses to address potential influences of protocol adherence, missing data, and the bounded nature of the measurement scale. First, a per-protocol analysis was performed on participants with an intervention adherence of at least 70%. Next, a complete-case sensitivity analysis was conducted with 71 participants to ensure findings were not skewed by specific exclusions. Furthermore, to account for the 0–100% bounded scale of intelligibility, a logit-transformed ANCOVA was utilized. We also employed Quade’s test (ranked ANCOVA) to address potential non-normality at baseline.

The per-protocol analysis yielded an adjusted mean difference of 3.57 (95% CI −1.79 to 8.92; *P*=.19), which was directionally consistent with the primary results. Notably, the adjusted group difference remained significant even when stroke phase was included as an additional covariate (mean difference 4.51, 95% CI 0.71–8.31; *P*=.02), which suggested the main effect was stable regardless of recovery stage. While the logit-transformed ANCOVA showed a marginal group effect on the logit scale (estimated difference 0.52, 95% CI −0.01 to 1.05; *P*=.05), the overall inference remained consistent with the primary analysis.

Table S1. Supplementary analyses assessing robustness of treatment effects^a-e^

| Analysis | Estimated difference | *F*^b^ | *P*^c^ | *η²*^d^ |
| --- | --- | --- | --- | --- |
|  | Mean (95% CI^a^) |  |  |  |
| Per-protocol analysis | 3.57 (−1.79 to 8.92) | 1.80 (1, 42) | .19 | .04 |
| Complete-case analysis (n=71) | 4.41 (0.62 to 8.20) | 5.31 (1, 67) | .02 | .07 |
| Adjusted for stroke phase | 4.51 (0.71 to 8.31) | 5.60 (1, 68) | .02 | .08 |
| Ranked ANCOVA (Quade’s test) | 3.39 (−4.47 to 11.26) | 0.74 (1, 69) | .39 | .01 |
| Logit-transformed ANCOVAᵉ | 0.52 (−0.01 to 1.05) | 3.90 (1, 69) | .05 | .05 |

^a^CI, confidence interval

^b^*F*: F statistic

^c^*P*: P value

^d^*η*², partial eta squared (effect size estimate)

^e^Estimates are on the logit scale (intelligibility percentage transformed to logit after small offset); inference was consistent with the primary analysis

### 2. Stroke Phase-Stratified Analysis

As an exploratory measure, participants were stratified into three stroke phases (acute, early subacute, and chronic) to examine whether treatment effects varied by recovery stage. A factorial ANCOVA was conducted with post-intervention intelligibility as the dependent variable. The model included treatment group and stroke phase as fixed factors, while adjusting for baseline intelligibility and age as covariates.

In the early subacute phase, post-intervention intelligibility was significantly higher in the intervention group than in the control group (adjusted mean difference 8.79, 95% CI 2.52 to 15.06; *P*=.01). No significant between-group difference was observed in the acute phase, where wide confidence intervals reflected the small sample size (n=9). In the chronic phase, the adjusted mean difference favored the intervention but did not reach statistical significance (adjusted mean difference 4.13, *P*=.12).

Table S2. Phase-stratified ANCOVA results by stroke phase

| Stroke phase | Adjusted mean differenceᵃ | 95% CI | *P* |
| --- | --- | --- | --- |
| Acute (n=9) | −5.45 | −15.94 to 5.04 | .30 |
| Early subacute (n=28) | 8.79 | 2.52 to 15.06 | .01 |
| Chronic (n=36) | 4.13 | −1.10 to 9.37 | .12 |

ᵃAdjusted mean differences estimated using an ANCOVA model adjusting for baseline intelligibility and age. Positive values indicate higher post-intervention intelligibility in the intervention group.

### 3. Exploratory Analyses Related to Age Adjustment

To address the age imbalance observed at baseline, exploratory analyses were conducted to verify the directionality of the age coefficient. This was intended to explain the slight change in the adjusted between-group difference after including age in the primary ANCOVA model. The analysis involved univariable, baseline-adjusted, and multivariable models to observe how age interacted with other predictors.

Age was found to be significantly correlated with baseline intelligibility (*r*=-0.21, *P*=.04). In the univariable model, age was a significant predictor (*B*=0.35, *P*=.02), and this significance remained when adjusted only for baseline intelligibility. However, in the full multivariable model including the treatment group, the effect of age became non-significant (*B*=0.03, *P*=.73), while the treatment group effect remained significant (*P*=.02). These results clarify that age's influence on the model was largely accounted for by its relationship with baseline scores. Results are summarized in Table S3.

Table S3. Exploratory analyses examining age adjustment

| Analysis set | Predictors | Key results |
| --- | --- | --- |
| Univariable | Age | Age: *B*=0.35, *P*=.02 |
| Baseline-adjusted | Age, Baseline speech intelligibility | Age: *B*=0.23, *P*=.02; Baseline intelligibility: *B*=−0.41, *P*<.001 |
| Multivariable | Treatment group, Baseline speech intelligibility, Age | Baseline intelligibility: *B*=−0.26, *P*<.001; Age: *B*=0.03, *P*=.73; Treatment group: *P*=.02 |
| Correlation | Age vs Baseline speech intelligibility | Pearson *r*=−0.21, *P*=.04 |

Change in intelligibility was defined as post-intervention minus baseline score (positive values indicate improvement). “Baseline intelligibility” denotes the baseline speech intelligibility score. Treatment group was entered as an indicator variable (intervention vs control). Coefficients (B) are unstandardized regression coefficients.

### 4. Assay Sensitivity and Robustness Analysis

To address concerns about the clinical relevance of the findings, we conducted an analysis based on within-group changes and responder proportions using the ITT set. Responder thresholds were set at ≥7 and ≥15 points to contextualize gains against minimal clinically important difference ranges cited in the literature.

Within-group changes in intelligibility were significant for both the intervention group (mean 11.60) and the control group (mean 7.17), with P < .001 for both. While the proportion of responders at the ≥7-point threshold was similar between groups (52.6% vs 54.3%), the intervention group showed a higher percentage of responders at the ≥15-point threshold (28.9% vs 14.3%), although this difference was not statistically significant (*P*=.16). Results are presented in Table S4.

Table S4. Assay sensitivity and robustness analysis

Panel A. Assay sensitivity and robustness analysis (Intention-to-treat set)

| Analysis | Intervention | Control | P value |
| --- | --- | --- | --- |
| **Within-group change in intelligibility (post–baseline), mean (95% CI)** | 11.60 (8.06 to 15.14) | 7.17 (4.70 to 9.63) | <.001 for both groups |
| **Responders (≥7-point improvement), n/N (%)** | 20/38 (52.6) | 19/35 (54.3) | Fisher’s exact *P*=1.00 |
| **Responders (≥15-point improvement), n/N (%)** | 11/38 (28.9) | 5/35 (14.3) | Fisher’s exact *P*=0.16 |

Panel B. Robustness analysis (per-protocol set)

| Analysis | Adjusted mean difference, 95% CI | P-value |
| --- | --- | --- |
| Per-protocol (≥70% adherence) | 3.57 (-1.79 to 8.92) | .19 |

### 5. Statistical Diagnostics and Distribution

Diagnostic plots, including a Q–Q plot of standardized residuals and a residuals-versus-fitted scatter plot, were examined to verify the assumptions of the primary ANCOVA model. Additionally, histograms of baseline and post-intervention scores were plotted with a 95% vertical reference line to visualize potential ceiling compression.

The diagnostic plots did not reveal marked deviations from normality or homoscedasticity. The median baseline score was 89.00, and only one participant (1.4%) reached the ceiling level of ≥95 at baseline. This indicates that the primary model assumptions were met and that ceiling effects did not substantially bias the results.


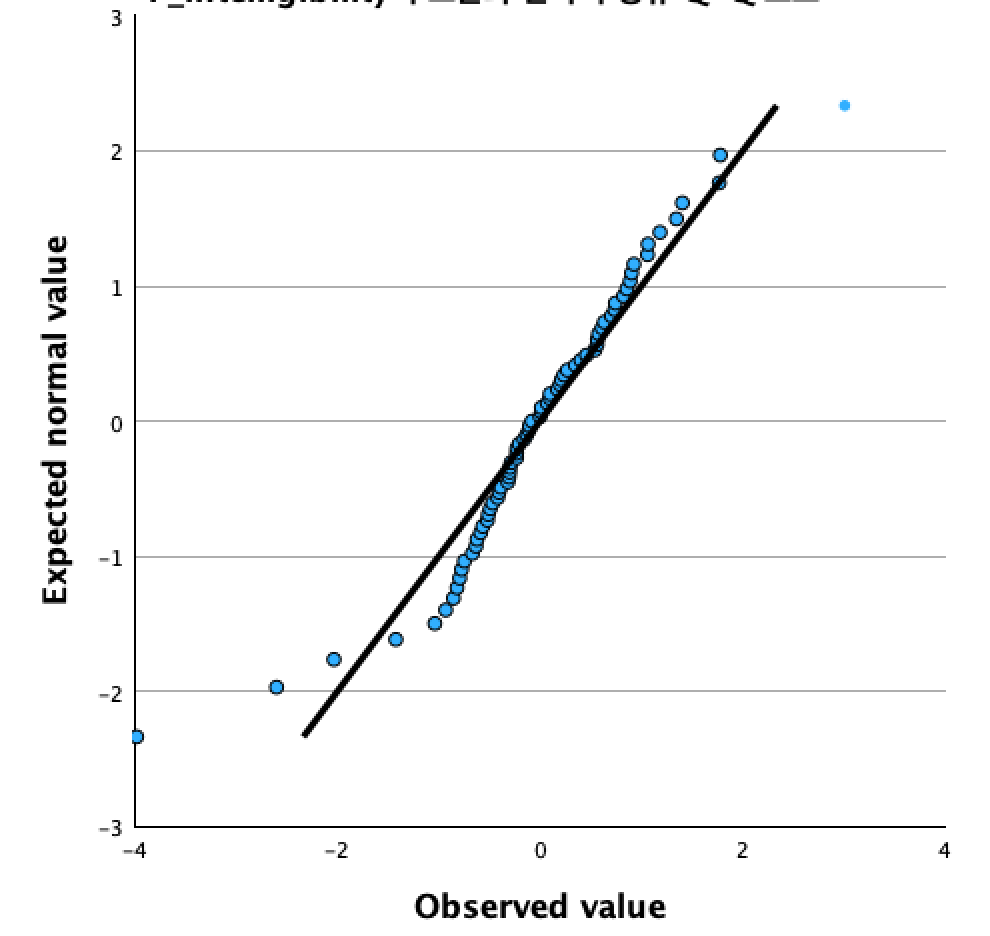


Figure S3. Normal Q–Q plot of standardized residuals from the primary ANCOVA model.


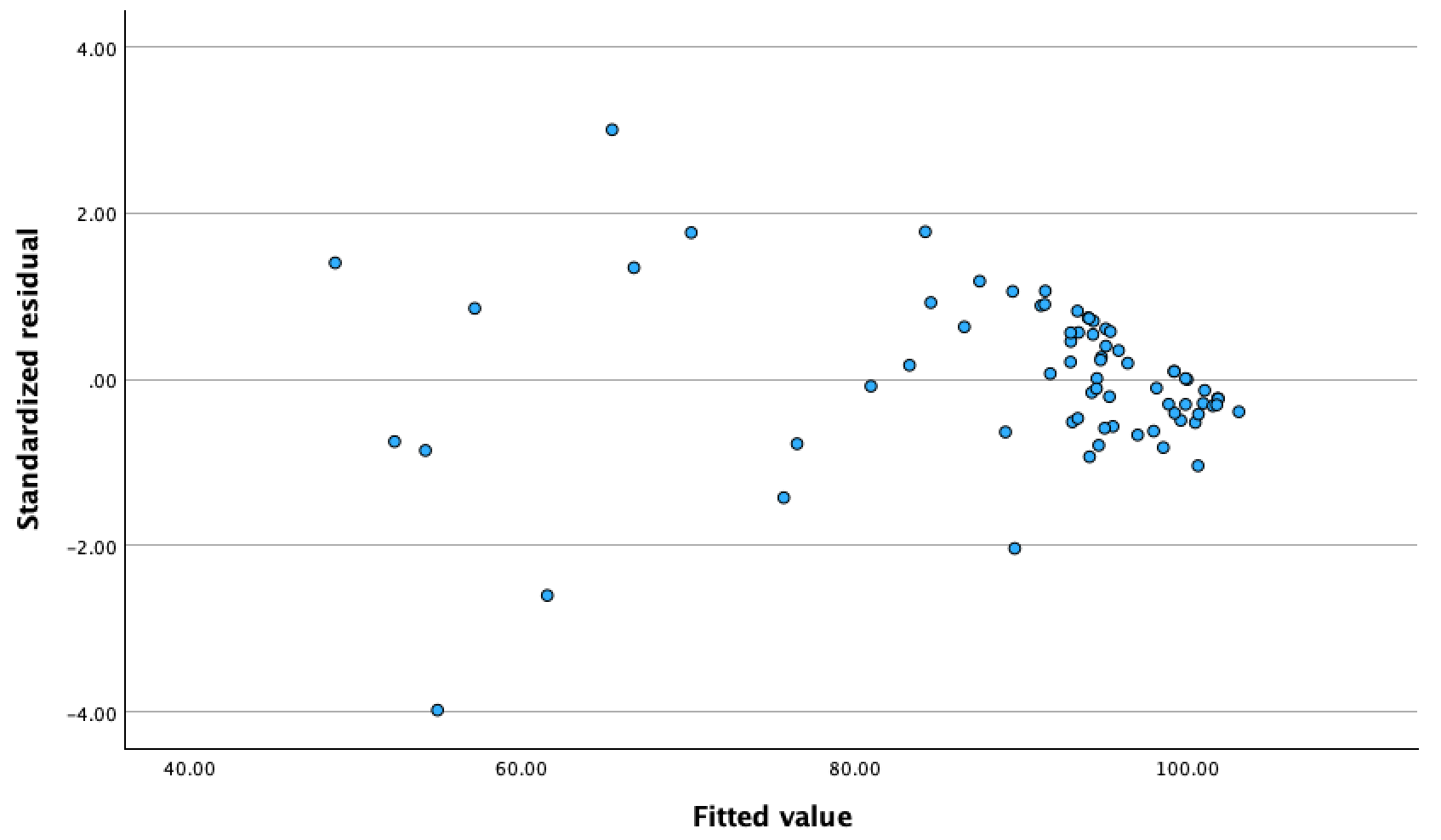


Figure S4. Standardized residuals versus fitted values from the primary ANCOVA model.

To visualize potential ceiling compression, we plotted a histogram of baseline intelligibility with a vertical reference at 95%. Ceiling counts were defined as the number and percentage of participants scoring ≥95%. The median baseline score was 89.00 (77.67–90.67), and one participant reached the ceiling level.


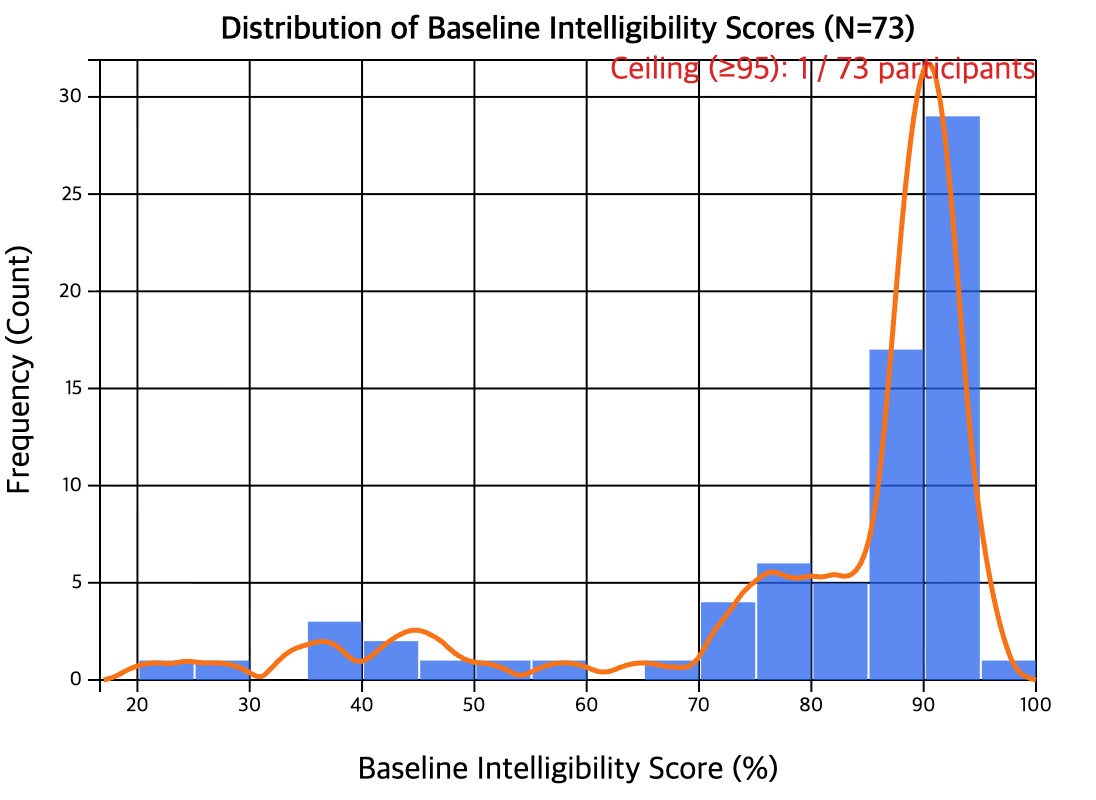


Figure S5. Distribution of baseline speech intelligibility scores (N=73).
The red vertical line marks the 95% threshold for potential ceiling effects.
